# Competence of healthcare professionals performing electroencephalography test: A systematic review

**DOI:** 10.1016/j.cnp.2025.03.001

**Published:** 2025-03-08

**Authors:** Elina Linnavuori, Irina Virtanen, Minna Stolt

**Affiliations:** a Department of Nursing Science, University of Turku, Turku, Finland; b Department of Clinical Neurophysiology, Turku University Hospital, Finland; c Wellbeing Services County of Satakunta, Pori, Finland; d University of Turku, Turku, Finland

**Keywords:** Diagnostic testing, Electroencephalography, Competence, Nursing, Allied healthcare

## Abstract

**Objective:**

To describe the EEG competence of healthcare professionals and how this competence has been measured in previous literature.

**Methods:**

A systematic review following the preferred Reporting Items for Systematic Reviews and Meta-Analyses. A literature search was conducted in CINAHL, PubMed, Scopus, and Web of Science databases focusing on studies that empirically examined the EEG competence of healthcare professionals.

**Results:**

A total of 28 studies were included. EEG competence consists of two main categories: knowledge and skills of EEG, and attitudes and values towards EEG. The EEG competence of healthcare professionals was assessed in three different settings: tests, simulations, and real life. The data collection methods were knowledge tests, self-assessments, and observations. The tools were developed by a researcher(s) for the single study and were not psychometrically tested.

**Conclusion:**

EEG competence is a multidimensional concept that includes knowledge, skills, attitudes, and values that need to be considered when defining EEG competence and developing tools to measure it.

**Significance:**

This systematic review provides information to the educators of healthcare professionals and healthcare organizations involved in developing comprehensive EEG training programs and assessments to foster professional development and ensure reliable diagnostic test results for patients.

## Highlights

• A comprehensive and multi-method assessment tool to measure EEG competence is needed.
• Recent research on the EEG competence of healthcare professionals is scattered.
• Performing EEG is within the scope of practice of many healthcare professionals with varying educational backgrounds.

## 1 Introduction

Electroencephalography (EEG) is a diagnostic test and most often, it is used for diagnosing epilepsy (Tatum et al., 2018). EEG is also a valuable tool to detect and evaluate the treatment of non-convulsive status epilepticus (NCSE) or non-convulsive seizure (NCS), identification of ischemia, and give information on prognostication for critically ill patients (Tatum et al., 2018). Performing EEG is within the scope of practice of many healthcare professionals with different education backgrounds. Mostly allied healthcare professionals such as technicians or technologists (Cole et al., 2022) but also increasingly a responsibility of nursing professionals (Taran et al., 2021), mostly in intensive care settings. This is due to the increasing number of EEG monitoring required and the fact that EEG professionals are often not available outside of office hours (Taran et al., 2021). In this review, we use the term healthcare professionals to cover all those professionals who are expected to perform EEG tests in different healthcare settings.

EEG is available in both peripheral and tertiary centers (Cole et al., 2022), and different EEG methods are used in both outpatient and inpatient settings, these include: standard outpatient EEG performed in a laboratory setting, ambulatory EEG recorded outside the hospital with a portable device, long-term video EEG monitoring over several days to record typical patient seizures, and continuous EEG recording, especially in critically ill patients (Tatum et al., 2018). To provide accurate and reliable test results in a variety of settings, healthcare professionals who are expected to undertake an EEG need specific competence in handling the testing process. They are on the scene to identify and address potential sources of error and to observe the patient from the bedside. Poor technical quality of an EEG may not allow conclusions to be drawn or may lead to misinterpretation and misdiagnosis (Tatum et al., 2018), for example, if electrodes are placed incorrectly or artifacts are not identified or corrected during recording.

In this review, the EEG testing process is described following the work process of medical imaging (Balogh et al., 2015) and focuses on the pre-analytical, analytical, and post-analytical phases. This is due to the assumption that nurses, technicians and technologists are specifically focused on these phases in the diagnostic testing process. An EEG test starts with the pre-analytical phase, which includes preparing the patient for the test, e.g., informing and instructing the patient about the test (Rayi and Murr, 2022), and placing the electrodes on the patient (Seeck et al., 2017, Sinha et al., 2016). This is followed by the analytical phase which includes performing the test on the patient e.g., following the test procedure according to the guidelines, taking care of the patient, and providing a preliminary analysis (Sinha et al., 2016, ASET 2021)—finally, the post-analytical phase includes interpreting the test result and providing a reporting (Sinha et al., 2016). In this phase, interpretation and reporting are typically carried out by clinical neurologists or neurophysiologists (Cole et al., 2022).

The practices in EEG are guided by different competency requirements (e.g. American Society of Electroencephalographic Technicians, ASET – The Neurodiagnostic Society, ASET 2021) and guidelines or consensus statements regarding different EEG methods for different patient groups or healthcare settings (e.g. Peltola et al., 2023, Kuratani et al., 2016, Herman et al., 2015).

The concept of EEG competence lacks international consistency. In this review, the focus is on the generic EEG competence that is expected from everyone in the reference group who performs EEG and covers all EEG methods in different settings. Therefore, EEG competence is viewed according to McMullan et al. (2003) “*job-related, being a description of an action, behaviour or outcome that a person should demonstrate in their performance*” as distinct from the term competency, which emphasizes the more advanced performance of an individual conducting a process (McMullan et al., 2003). In the holistic approach, competence consists of different attributes such as knowledge, skills, attitudes, and values (Fukada, 2018). In this review competence is defined as “the sufficient ability to combine knowledge, skills, attitudes, and values to cope with specific practical situations” (Meretoja et al., 2004), which refers here to the EEG process of diagnostic testing.

As the competence of healthcare professionals is important to ensure high-quality EEG recordings (Tatum et al., 2018), a comprehensive study focusing on the assessment of EEG competence is needed. Recently, two reviews have been published to discover what kind of education programs are offered to healthcare professionals who do not have previous EEG training or education (Kromm et al., 2021, Taran et al., 2021). These reviews did not focus on assessing EEG competence or identifying the components of EEG competence. In these reviews, the search was also limited to specific patient groups, such as adult EEG (Kromm et al., 2021), or contexts, such as intensive care environments (Taran et al., 2021). Based on these results there is a need to understand the EEG competence of healthcare professionals more comprehensively to ensure patient safety and prevent diagnostic errors. This systematic review was conducted to search the existing literature to synthesize the evidence on measurements of the overall EEG competence of healthcare professionals based on previous empirical studies.

## 2 Aims

This systematic review aims to describe the EEG competence of healthcare professionals and how this competence has been measured in previous literature. The results can be used to obtain information on EEG competence and assessment needs.

Review questions were:

1. What are the components of EEG competence of health care professionals that have been measured in previous empirical studies?
2. What factors, if any, are associated with EEG competence of healthcare professionals?
3. How has the EEG competence of healthcare professionals been measured?

## 3 Methods

### 3.1 Design

A systematic review was conducted following a prior planned but unpublished review protocol. The findings are reported according to the Preferred Reporting Items for Systematic Reviews and Meta-Analyses (PRISMA) (Page et al., 2021).

### 3.2 Search

A comprehensive electronic literature search was conducted on 24 January 2023 in CINAHL, PubMed, Scopus, and Web of Science databases. Search terms were generated using the Boolean operators AND, OR, and MeSH keywords. The search terms were (nurse* OR technician* OR technologist* OR “biomedical laboratory scien*”) AND (EEG OR electroencephalog*) AND (skill* OR competen* OR know* OR abilit* OR capabilit* OR perform* OR practic* OR attitude* OR value* OR interpret* OR detect OR experience*). MeSH keywords “Electroencephalography” and “Professional Competence” were used in CINAHL and PubMed databases.

### 3.3 Eligibility criteria

The search strategy was to locate articles with different study designs that had empirically examined the EEG competence of healthcare professionals who are expected to perform EEG tests in different healthcare settings such as: nurses, technologists, technicians, and biomedical laboratory scientists or students. Only articles that focused solely on EEG competence were included. Therefore, articles that *focus only on physicians’ EEG competence,* and articles where *EEG was only one part of the assessment of nursing competence* were excluded. We included articles that were published in English in peer-reviewed journals. The context for this review was not limited to any specific healthcare setting. In addition, no time limitations were set. Table 1.

**Table 1:** Inclusion and Exclusion Criteria.

*Inclusion Criteria*	*Exclusion Criteria*
Empirical studies that focus on Health care professionals’ EEG competence	Textbook chapter, editorial, guidelines, theoretical and opinion papers, consensus statements, standards, concept analyses, systematic reviews, conference reports
measurement of EEG competence is included	
quantitative EEG, raw EEG	processed EEG only, e.g., bispectral index (BIS)
healthcare professionals such as nurses, technologists, technicians, and biomedical laboratory scientists who perform EEG and students of these	physicians EEG competence
published in English in peer-reviewed journals	

### 3.4 Selection of sources of evidence

All identified relevant records were imported into Zotero, and duplicates were removed. Titles and abstracts were screened by two authors (EL, IV) for assessment against the inclusion criteria for the review. Potentially relevant sources were retrieved in full. Full texts that did not meet the inclusion criteria were excluded. Disagreements between the reviewers were resolved through discussion. In case of disagreement, a third author (MS) participated in the decision-making process. The final inclusion or exclusion was confirmed by the whole research group.

### 3.5 Data extraction and synthesis

The first author extracted the data from the included studies using a data extraction sheet developed for the purposes of the review. The data extraction sheet included the following details: the author(s), year of publication, location, purpose of study, participants, context, methodology, EEG competencies, how EEG competencies were measured, and factors associated with EEG competence.

### 3.6 Data analysis

Studies were analyzed using both qualitative inductive and deductive content analysis (Kyngäs et al., 2019) focusing on the manifest content; no interpretation was made (Kyngäs et al., 2019, Graneheim and Lundman, 2004). First, in the preparation phase meaning units as words and sentences were identified that answered the research questions (Kyngäs et al., 2019). The identified meaning units were condensed and labeled with a code. (Graneheim and Lundman, 2004.) Second, in the organization phase, codes were grouped based on the similarities to form subcategories which were further sorted into main categories (Kyngäs et al., 2019).

Regarding the first research question, the deductive content analysis was based on the competence definition used (Meretoja et al., 2004). The deductive categorization matrix includes knowledge and skills of EEG and attitudes and values towards EEG. Codes that describe EEG competence with different competence attributes were organized under these categories. After this, the inductive content analysis continued with the codes being grouped into sub-categories based on similarities that formed the EEG competence components.

The quality and bias of each included studies in this review was assessed by one of the authors using the Mixed Methods Appraisal Tool (MMAT) (Hong et al., 2018). MMAT tool consists of two screening questions for all types of studies. The other five criteria were rated from categories based on the research designs of the included studies: Qualitative (n = 1), Quantitative non-randomized (n = 25), and Quantitative descriptive studies (n = 2). No studies were excluded based on the quality assessment.

## 4 Results

### 4.1 Characteristics of sources of evidence

**Fig. 1:**
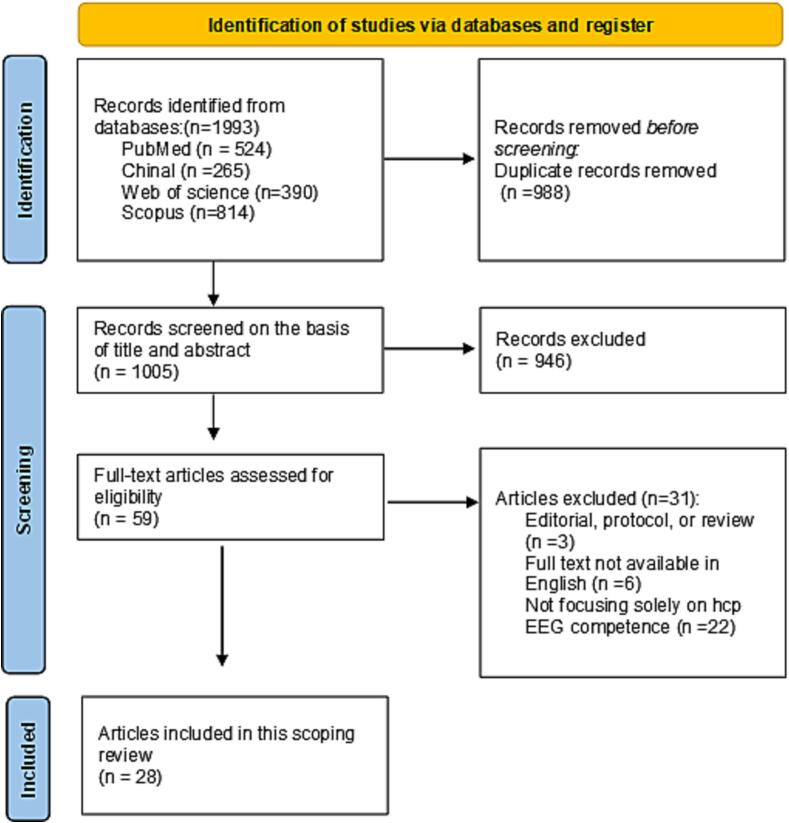
Flow diagram of study selection (Page et al., 2021).

A total of 1993 records were identified from the databases. After removing duplicates, 1005 studies were screened based on the title and abstract level. Of those 59 potentially relevant full-text articles were assessed for eligibility. A total of 28 studies met all the inclusion criteria and were included in the systematic review. Fig. 1.

The studies included in the review were published between 1996 and 2022. The majority being from the last ten years. The studies were conducted in the USA (n = 12), Canada (n = 3), Australia (n = 2), Finland (n = 2), France (n = 2), England (n = 2), South Africa (n = 1), Germany (n = 1), Singapore (n = 1), and Turkey (n = 1). Of the studies, 15 out of the 28 studies included different educational interventions mostly using pre and post-test designs. The other study designs used were prospective studies (n = 4), and a cross-sectional study (n = 1). Eight studies did not specify the study design. Table 2.

**Table 2:** Characteristics of the studies (n = 28).

Author(s), year of publication, location	Aim	Study design	Data collection (setting and assessment method)	Participants	Clinical setting	Quality appraisal (MMAT)
Ahrens et al., 2021 U.S	To assess the interrater agreement, sensitivity, and specificity of EEG technologists for detecting seizures	Not reported,	Test setting	n = 32	Pediatric epilepsy center	5/7
			Knowledge test:	29 EEG technologists		
			−90 five-minute sample pediatric video EEG with multiple choice responses	3 clinical neurophysiologists		
Amorim et al., 2017 U.S	To investigate the ability of ICU nurses to use CSA to identify seizures after a training session	A prospective, cross-sectional study	Test setting	n = 37	Adult Neurocritical care unit	5/7
		−post-test	Knowledge test:	33 Neuro-ICU nurses		
			−40 different CSA images: indicate whether any seizures were present and seizure count using a ratio scale.	4 neurophysiologists		
Asukile et al., 2022 South Africa	To determine the effectiveness of EEG teaching program (EEGonline) in improving EEG analysis and interpretation skills	A prospective cohort study	Test setting	n = 91	Not reported	7/7
		−pre-test	Knowledge test:	74 neurologists,		
		−post-test	−30 questions, each comprising 1 or more EEG waveforms of interest requiring identification multiple choice responses	4 other medical practitioners (neurology residents)		
			Self-assessment:	13 medical technologists		
			−6 matrix-type questions: satisfaction with the course and confidence in analyzing various EEG waveforms.			
Beirne et al., 1996 UK	To compare medical histories written by clinicians and EEG technicians	A prospective study	Real-life setting	n = not reported	Child	6/7
			Indirect observation:	clinicians and EEG technician	A routine out-patient EEG	
			−Prospective evaluation for a two-month period of the histories obtained by both the clinician and the EEG technician			
Beuchat et al., 2021 Germany	To investigate the interrater agreement (IRA) between EEG technologists and certified neurophysiologists. Additionally, to examine any clinical factors that might influence it.	A single-institution prospective cohort study	Real-life setting	n = 13	Adults and adolescents (≥ 15 years old)	6/7
			Indirect observation:	8 EEG technologists	A routine EEG	
			−Interpret EEG at the bedside using the EEG interpretation sheet and analyze different EEG parameters using yes/no questions	5 certified neurophysiologists		
Björn et al., 2020 Finland	To examine the development of biomedical laboratory scientist students’ theoretical knowledge and practical skills in the EEG measurement when using a virtual EEG simulator	Mixed-methods intervention study	Test setting	n = 35 biomedical laboratory science students	A clinical neurophysiology course	4/7
		−pre-test	Knowledge test:			
		−post-test	−8 specific questions of the EEG method for electrode placement and neurophysiology theory using multiple-choice responses			
			Simulated setting			
			Direct observation:			
			−Practical hands-on evaluation of EEG electrode placement using two sets of assessment guidelines			
Bourgoin et al., 2020 France	To evaluate the ability of bedside critical care providers to detect abnormal traces in aEEG after its implemented into nursing care.	A prospective study	Real-life setting	n = 51 pediatric intensive care unit nurses	Pediatric intensive care unit	5/7
		−post test	Indirect observation:		PICU	
			−Participant was in charge of a patient for a maximum shift of 12 h and completed a summary form related to shift			
Dericioglu et al., 2015 Turkey	To revisit the use of DTA (digital trend analysis) methods for seizure detection by non-expert physicians, especially nurses working in intensive care units	Cohort study	Simulated setting	n = 4	Adult neurological ICU (NICU)	6/7
			Indirect observation:	1 critical care neurology fellow		
		−post test	−Twenty traces from 10 patients and 10 controls in random order.	1 neurology resident		
			−Participants marked seizures by a moving cursor that indicated the exact time point as it was scrolled back and forth on the screen.	2 NICU nurses		
Du Pont-Thibodeau et al., 2017 U.S	To determine the accuracy and confidence of CCM providers to identify a seizure using amplitude-integrated EEG (aEEG) and aEEG combined with Color Density Spectral Array Electroencephalography (aEEG + CDSA) after a tutorial	Tutorial and questionnaire	Test setting	n = 23	Pediatric ICU	5/7
		−post-test	Knowledge test:	6 attending physicians		
			−200 aEEG and aEEG + CDSA images classification of the image as having a seizure(s) or no seizure.	12 fellows		
			Self-assessment:	5 nurses		
			−Level of confidence in interpreting aEEG and aEEG + CDSA images	in the pediatric intensive care unit		
Lalgudi Ganesan et al., 2018 Canada	To compare the performance of critical care providers and EEG experts in identifying seizures using QEEG tools after intervention and to try to determine the factors influencing this.	Post-test	Simulated setting	n = 12	Pediatric ICU	6/7
			Indirect observation:	3 ICU fellows		
			−27 continuous electroencephalograms where participants mark any epochs that they “suspected to be seizures” using a single-pixel cursor on the actual QEEG trend	3 ICU nurses,		
				3 neurophysiologists		
				3 electroencephalography technologists.		
Gilbert, 2000 England	To evaluate nurses' ability to identify seizures and types of Status epilepticus (SE) after training	A pretest–posttest design	Test setting	n = 15 registered nurses	Adult epilepsy unit	6/7
			Test:	with at least 2 years of neuroscience nursing experience and special education		
			−5 video examples in which the participant had to identify the type of SE the patient was experiencing			
			Self-asessement:			
			−Confidence (4 items) in identifying status types and perception of the usefulness of the algorithm, Likert scale 1–5 1 (not confident) to 5 (very confident)			
Goswami et al., 2018 Canada	To establish uniformity of care in 2 level III neonatal intensive care unit (NICU) and provide standardized brain monitoring 24/7 for eligible newborns.	Not reported	Real-life setting	n = not reported,	Neonatal intensive care units (NICU)	6/7
			Indirect observation:	neonatal intensive care unit (NICU) nurses		
			−Neonatal nurses apply scalp electrodes, troubleshoot technical issues, and identify amplitude-integrated EEG abnormalities.			
			− A cohort of 100 infants with moderate to severe hypoxic-ischemic encephalopathy before and after the training program was compared.			
Kaleem et al., 2021 U.S	To determine neuroscience intensive care unit (neuro-ICU) nurse performance in interpreting quantitative EEG (qEEG) for seizure detection at the bedside.	A single-institution prospective cohort study	Real-life setting	n = 65	Adult intensive care unit (ICU)	6/7
			Indirect observation:	neuroscience intensive care unit nurses		
			−For the duration of their shift, the nurses logged the number of seizures: no seizures, 1–2 seizures, 3–5 seizures, 6–10 seizures, or > 10 seizures.			
Kang et al., 2019 U.S	To evaluate neuro-ICU nurse performance using real-time bedside qEEG interpretation to detect recurrent nonconvulsive seizures.	A prospective, single-institution study	Real life setting	n = not reported,	Adult Neuro-ICU.	4/7
			Indirect observation:	Neuro-ICU nurses		
			− For the duration of the shift, the nurses logged the number of seizures: no seizures, 1–2 seizures, 3–5 seizures, 6–10 seizures, or > 10 seizures.			
Kolls et al., 2012 U.S	To determine whether healthcare providers who are not certified as EEG technologist can produce an EEG signal of the same quality using the 10/20 system-based template (BraiNet) as certified technologists using the 10/20 system.	Not reported	Real-life setting	n = not reported,	Adult Neurocritical care unit	4/7
			Indirect observation:	health care providers that have not been certified as EEG technologists,		
			−nurses applaid EEG leads using the BraiNet template system	and		
				EEG technologists		
Legriel et al., 2021 France	To evaluate the effectiveness of an training program designed to teach basic interpretation of the cEEG to critical care staff.	observational study	Test setting	n = 108 ICU staff professions:	Adult ICU	5/7
		A Prospective Multicenter Study	Knowledge test:	15 Senior intensivist		
		pre-test	−(10 questions)	12 Fellow		
		post-test	− 23-s EEG images (epoch longitudinal bipolar 8-channel) using simple choice and multiple-choice questions	23 Resident		
		1 day		19 Medical students		
		15 day		39 Nurse		
		30 day				
		90 day				
Leira et al., 2004 U.S	To assess the ability of various non-expert bedside caregivers to recognize epileptiform discharges.	A prospective cohort study with an educational intervention	Test setting	n = 50	Adult ICU	6/7
		pre-test	Knowledge test:	15neurology residents,		
		post-test	−24 raw EEG images (10-s sample of 16-channel adult EEG recording).	3EEG technician 3neurosurgery residents,		
			− recognize epileptiform discharges using yes/no questions	8 neurology floor nurses,		
				3 medical ICU fellows		
				12 neurological icu nurses		
				6 medical icu nurses		
Linnavuori et al., 2022 Finland	To analyze subjectively	A descriptive cross-sectional study	Test setting	n = 65 healthcare professional who performs EEG:	Rutin EEG	5/7
	and objectively assessed EEG competence of healthcare professionals		EEG Competence instrument (EEGcomp):	34 registered nurses		
			−Self-assessed EEG competence on a 5-point Likert scale (54 items)	31 laboratory technologists		
			−The knowledge test section, (6 items) using filling-in-the-blank and multiple-choice questions			
Mehta et al., 2017 Australia	To evaluate inter-observer reliability in interpreting aEEG recordings	Not reported	Simulated setting	n = 3 NICU clinicians with varying levels of experience in interpreting aEEG:	Neonatal intensive care unit	6/7
			Indirect observation:	a neonatologist	NICU	
			−Observers rated aEEG traces in the digital review software using a standardized score sheet	a nurse educator in neonatology		
				a clinical nurse specialist in neonatology		
Ouchida et al., 2022 Australia	To investigate how effectively the designed clinical testing tool is used during ictal and postictal periods, its performance rate and nurses' knowledge of clinical testing.	Not reported	Test setting	n = 47	Adult epilepsy monitoring unit/neurology unit	5/7
			Knowledge test:	37 neurology unit nurses		
			−10 multiple-choice knowledge questions	10 NNTs (neurophysiology nurse technologist = registered nurses who were also designated as NNTs->worked at the clinic as EEG technologists)		
			Self-assessment:			
			− nurse confidence testing the patient using a Likert scale			

			Real-life setting			
			Indirect observation:			
			− Patient Clinical Testing during a seizure			
Picinich et al., 2020 U.S	To evaluate if an educational program can be used to teach nurses how to monitor continuous electroencephalography (cEEG), recognize a burst suppression pattern, and quantify the duration of suppression. This aims to establish their competency in adjusting sedative infusions to achieve a burst suppression goal.	A pre-post evaluation study	Test setting	n = 13 Neuroscience intensive care unit (NSICU) nurses	Neuroscience ICU	5/7
		pre-test (PT-0)	Knowledge test:			
		post-test	−15 multiple-choice questions that included references to raw EEG data			
		PT-1 month	Self-assessment:			
		PT-3 month	−Current level of comfort with cEEG monitoring using a 0–10 scale			
		PT-6 month				
Poon et al., 2015 Singapore	To assess how e-learning approach teaches amplitude-integrated electroencephalography (aEEG).	a cross-sectional study	Test setting	n = 37 NICU staff:	neonatal intensive care units	6/7
		pre-test	The written survey:	32 nurses	NICU	
		post-test	(25 questions)	5 doctors		
			Self-assessment:			
			−confidence on interpreting aEEG, a scale 1–10			
			Knowledge test:			
			−5 objective questions testing the participant’s ability to correctly identify aEEG tracings			
Prendergast et al., 2022 U.S	To describe the duration from when nurses identify electrographic seizures to the initiation of seizure treatment.	Not reported	Real life setting	n = 24 nursing staff responded to the survey to assess nurses’ comfort with QEEG	Pediatric intensive	5/7
			Indirect observation	n = 44 EEGs (30 patients) forms were evaluated for nurses’ recognition of electrographic seizures	care unit	
			−nurses track information on a standardized data collection form to while the QEEG was in place			
			Test setting			
			Self-assessmet:			
			nurses’ comfort with QEEG at the bedside			
Seiler et al., 2012 U.S	To evaluate the effectiveness of a staff	A quasi-experimental pretest/posttest 1-group design	Test setting	n = 47 Neuroscience ICU registered nurses	Adult NSICU.	5/7
	educational program designed to enhance nurses' knowledge in utilizing continuous electroencephalography (cEEG) monitoring	pre- test	Knowledge test:			
			−a 20- multiple-choice/true–false question			
		post-test 1				
		post-test 2				
Swarnalingam et al., 2022 Canada	To evaluate the sensitivity of nonconvulsive seizure detection using a panel of quantitative EEG (QEEG) trends	Single-centre, single-blinded observational study	Test setting	n = 11	Pediatric intensive care unit	6/7
			Knowledge test:	6 pediatric residents		
			−45 QEEG epochs containing 184 subclinical seizures were distributed through an online image-sharing and annotation tool Collabshot—Toptal LLC.	5 pediatric intensive care unit nurses		
			−Participants were asked to make a green tick mark on top of the area believed to depict an electrographic seizure	PICU		
Swisher et al., 2015 U.S	To evaluate the sensitivity and specificity of seizure detection using a panel of quantitative EEG (qEEG) trends	Not reported	Test setting	n = 17	Adult intensive care unit (ICU)	4/7
			Knowledge test:	5 neurophysiologists,		
			−qEEG panels (n = 180) (1 h each) to determine the number of seizures present in the panel: no seizures, 1 to 2 seizures, 3 to 5 seizures, 6 to 10 seizures or > 10 seizures.	7 EEG technologists		
				5 Neuroscience ICU nurses		
Topjian et al., 2015 U.S	To determine the accuracy and reliability of electroencephalographic seizure detection using color density spectral array (CDSA) electroencephalography (EEG) following a short training period	Tutorial and questionnaire	Test setting	n = 39 Critical care providers:	Pediatric ICU	5/7
		post-test	Knowledge test:	12 attending physicians		
			−200 CDSA images to identify seizures using yes/no questions	8 fellow trainees		
				19 nurses		
Yuan et al., 2022 U.S	To improve the practice of propofol-based total intravenous anesthesia (TIVA) by increasing the use of EEG guidance	Not reported	Test setting	n = 71	Pediatric anesthesia division.	5/7
			Knowledge test:	11 clinical fellows		
			−12- multiple-choice question	32 residents		
			theoretical and practical EEG knowledge	28 nurse anesthetists		

The target group of the studies varied. Fourteen of the studies included non-EEG-professionals e.g., intensive care unit nurses, with no previous experience of EEG while nine of the studies included EEG professionals such as EEG technicians or EEG technologists, with different levels of experience in EEG. Five studies include both. In one study, biomedical laboratory science students were the target group. Sixteen of the twenty-eight studies also included other participants such as neurophysiologists, neurologists, or ICU fellows. Neurophysiologists were often used as the “gold standard” in the study, with whom the results of other participants were compared. The sample sizes of these studies ranged from 3 to 108.

The setting of the studies was most often intensive care units (n = 17). Twelve of the included studies focus directly on adult EEG and eleven of the studies on pediatric EEG. Three of the studies did not specify the patient group. Most of the studies included in this review (n = 24) focused on the EEG interpretations made by healthcare professionals. Of these studies, most (n = 15) used quantitative electroencephalography such as color density spectra array (CDSA) or amplitude-integrated EEG (aEEG), and eight studies used raw EEG signals.

### 4.2 Quality of the included studies

MMAT tool was used to evaluate methodological quality and risk of bias of each study (Hong et al., 2018). The detailed quality evaluation results for each study are presented in supplementary file 1. The quality of the studies varied. Overall scores ranged from four to seven. All the included studies met the quality assessment criteria for the first two screening questions. Most of the included studies met at least 60 % of the other five criteria that were rated from categories based on the research designs of the included studies (Qualitative, Quantitative non-randomized, and Quantitative descriptive studies).

Methodological limitations were related to the description of the confounding factors, target population or sampling strategy, and the measurement methods causing some bias. The most common criterion the quantitative nonrandomized studies failed to meet was 3.4: “Are the confounders accounted for in the study design and analysis” causing a confounding bias because appropriate methods to control for confounders weren’t used. A sampling bias in quantitative non-randomized and quantitative descriptive studies was evident in some studies because there was no clear description of the target population. A measurement bias was caused in quantitative non-randomized and quantitative descriptive studies because the measurement methods were not validated, or reliability tested.

### 4.3 EEG competence of healthcare professionals

EEG competence was divided into two main categories: knowledge and skills of EEG and attitudes and values towards EEG. These main categories consist of seven subcategories that formed the components of EEG competence. Each EEG competence component was subdivided into more specific subcomponents according to the codes. These main categories and components of EEG competence are presented in Table 3 and described in detail below.

**Table 3:** EEG competence main categories, sub-categories, codes, and references.

Main categories	sub-categories	codes	References
1. Knowledge and skills of EEG	EEG interpretation	Artifacts	Bourgoin et al., 2020, Legriel et al., 2021, Linnavuori et al., 2022, Mehta et al., 2017, Picinich et al., 2020, Seiler et al., 2012
		Normal EEG	Asukile et al., 2022, Beuchat et al., 2021, Bourgoin et al., 2020, Legriel et al., 2021, Mehta et al., 2017, Picinich et al., 2020
		Epileptiform EEG	Ahrens et al., 2021, Amorim et al., 2017, Asukile et al., 2022, Beuchat et al., 2021, Bourgoin et al., 2020, Dericioglu et al., 2015, Du Pont-Thibodeau et al., 2017, Lalgudi Ganesan et al., 2018, Gilbert, 2000, Kaleem et al., 2021, Kang et al., 2019, Legriel et al., 2021, Leira et al., 2004, Prendergast et al., 2022
		EEG continuity	Ahrens et al., 2021, Mehta et al., 2017, Picinich et al., 2020, Poon et al., 2015, Seiler et al., 2012
		Effects of external factors	Legriel et al., 2021, Yuan et al., 2022
	EEG theoretical knowledge base	Epilepsy	Gilbert, 2000, Ouchida et al., 2022
		EEG Indications	Goswami et al., 2018, Ouchida et al., 2022, Picinich et al., 2020
		EEG terminology	Picinich et al., 2020
		Drug effects on EEG	Yuan et al., 2022
		EEG background	Björn et al., 2020, Linnavuori et al., 2022
	Management and use of medical devices	EEG electrodes	Seiler et al., 2012, Björn et al., 2020, Linnavuori et al., 2022, Kolls et al., 2012, Goswami et al., 2018, Bourgoin et al., 2020
		EEG device	Linnavuori et al., 2022, Goswami et al., 2018
	Collaboration	Information sharing	Goswami et al., 2018, Seiler et al., 2012
		Information gathering	Beirne et al., 1996
	Patient care	Patient observation	Linnavuori et al., 2022
		Safety issues	Ouchida et al., 2022
		Seizure management and assessment	Ouchida et al., 2022
2. Attitudes and values towards EEG	Confidence to act	Interpret EEG	Du Pont-Thibodeau et al., 2017, Poon et al., 2015, Asukile et al., 2022, Gilbert, 2000
		Testing patients during seizures	Ouchida et al., 2022
	Comfort with own EEG competence	Performing EEG	Picinich et al., 2020, Linnavuori et al., 2022
		Interpreting EEG	Prendergast et al., 2022
		Collaboration	Prendergast et al., 2022

#### 4.3.1 Knowledge and skills of EEG

The knowledge and skills concerning EEG that healthcare professionals were expected to have consisted of EEG interpretation (n = 23 studies), EEG theoretical knowledge base (n = 7 studies), management and use of medical devices (n = 6 studies), collaboration (n = 3 studies), and patient care (n = 2 studies).

***EEG interpretation*** consisted of five subcomponents that healthcare professionals should be able to detect or identify from the EEG signal: *artifacts*, *normal EEG* during wakefulness and sleep such as sleep–wake cycles (Bourgoin et al., 2020), common sleep figures (Beuchat et al., 2021), *epileptiform EEG* such as seizures (e.g., Ahrens et al., 2021), status epilepticus (e.g., Beuchat et al., 2021) or interictal epileptiform discharges (e.g., Leira et al., 2004), *EEG continuity* such as continuous/discontinuous (e.g., Mehta et al., 2017), burst suppression (e.g., Seiler et al., 2012), or, isoelectric EEG (e.g., Legriel et al., 2021), and/ or *effects of external factors* such as reactivity to auditory and nociceptive stimuli ( Legriel et al., 2021), or effects of sedation (Yuan et al., 2022).

***The EEG theoretical knowledge base*** consisted of four subcomponents: *necessary information about epilepsy*, such as seizures (Gilbert, 2000, Ouchida et al., 2022), and classification of epilepsy (Ouchida et al., 2022), *EEG indications, EEG terminology, effects of drugs on EEG*, and the *theoretical background of EEG.*

***The Management and use of medical devices*** consisted of two subcomponents about which healthcare professionals were expected to have knowledge and skills: *EEG electrodes* and *EEG devices*. Knowledge and skills about EEG electrodes included e.g., applying EEG electrodes according to the 10–20 system (Björn et al., 2020) or with the help of the BraiNet template (Kolls et al., 2012), as well as which leads are magnetic resonance imaging compatible (Seiler et al., 2012). Knowledge and skills relating to EEG devices included handling EEG devices e.g., marking clinical events, camera position, and troubleshooting equipment issues (Goswami et al., 2018).

***Collaboration*** included collaboration with patients, families, and other healthcare professionals and consisted of two subcomponents: *information sharing* e.g., the test result or certain problems (Goswami et al., 2018, Seiler et al., 2012), and adequate *information gathering* from patients (Beirne et al., 1996).

***Patient care*** consisted of three subcomponents: *patient observation, safety issues*, and *assessment and management of seizures* such as patient consciousness and language assessment and, performance on *testing patients during seizures* (Ouchida et al., 2022).

#### 4.3.2 Attitudes and values towards EEG

The attitudes and values towards EEG that healthcare professionals were expected to have consisted of two sub-categories: confidence to act and comfort with their own EEG competence.

***Confidence to act*** was perceived as being related to EEG interpretation and testing patients during seizures. ***Comfort with own EEG competence*** was perceived as being related to comfort with performing EEG, comfort with interpreting EEG, and comfort with collaboration.

### 4.4 Factors associated with the EEG competence of healthcare professionals

Some factors were identified that were significantly associated either positively or negatively with the subjectively and objectively assessed EEG competence of healthcare professionals. These factors were divided into two groups: individual-related factors (n = 3 studies) and work-related factors (n = 14 studies).

*The Individual-related factors* associated with EEG competence consisted of: age, work experience, own satisfaction with EEG competence, participation in out-of-work training, familiarization with EEG guidelines, reading EEG literature (Linnavuori et al., 2022) and EEG technologist certification (Ahrens et al., 2018). The factors that associated positively with self- assessed EEG competence were age, work experience, own satisfaction with EEG competence, participation in out-of-work training, and familiarization with EEG guidelines (Linnavuori et al., 2022). The factors that associated positively with the objectively assessed EEG interpretation were reading EEG literature (Linnavuori et al., 2022) and EEG technologist certification (Ahrens et al., 2018).

*The Work-related factors* associated with EEG competence consisted of targeted on-the-job training (Asukile et al., 2022, Björn et al., 2020, Gilbert et al., 2000, Legriel et al., 2021, Leira et al., 2004, Picinich et al., 2020, Poon et al., 2015, Dericioglu et al., 2015, Seiler et al., 2012), EEG signal characteristics (Kaleem et al., 2021, Kang et al., 2019, Swisher et al., 2015, Yuan et al., 2022), working hours (Kaleem et al., 2021), and patient characteristics (Beuchat et al., 2021). Targeted training was examined in ten studies using different educational interventions and comparing pre-and post-test scores. These targeted trainings significantly improved healthcare professionals' attitudes toward EEG (Picinich et al., 2020, Poon et al., 2015), as well as their knowledge and skills of EEG (Asukile et al., 2022, Björn et al., 2020, Gilbert et al., 2000, Legriel et al., 2021, Leira et al., 2004, Picinich et al., 2020, Poon et al., 2015, Dericioglu et al., 2015, Seiler et al., 2012, Yuan et al., 2022) Two studies revealed that certain EEG characteristics were more likely to be detected i.e. hemispheric seizures were more likely to be detected compared to focal seizures and longer seizures than briefer ones (Kaleem et al., 2021, Kang et al., 2019, Swisher et al., 2015). In addition, one individual study identified that patient characteristics including intubation, older age, known epilepsy history, and poor cooperation influenced the EEG interpretation (Beuchat et al., 2021). Inaccurate interpretation was associated with increased hours of EEG monitoring. (Kaleem et al., 2021.).

### 4.5 Methods to measure the EEG competence of healthcare professionals

Data were collected in three different settings: tests (n = 18 studies), simulations (n = 4 studies), and –real-life (n = 9 studies) using three types of data collection methods: self-assessment, knowledge test, and observation (direct or indirect). Three of the studies collected the data in two different settings. Moreover, nine of the studies used more than one method. Table 2.

The test settings measuring EEG competence of healthcare professionals used questionnaires. These questionnaires included knowledge tests and self-assessments. Simulated settings were used to assess EEG competence of healthcare professionals using indirect or direct observation. Real-life settings were used to assess healthcare professionals’ EEG competence in real workplace situations using indirect observation. The indirect observation included reviewing data sheets, monitoring patient’s electronic medical records or rechecking interpretation results focusing e.g. on the quality of the EEG signal, the time required to initiate a test, or the outcome of the patient.

Tools to assess healthcare professionals’ EEG competence were developed by the researcher(s) or research teams for each single study. Tests were based on the EEG guidelines of international societies (Linnavuori et al 2022) and EEG education materials (Yuan et al., 2022), current clinical evidence (Ouchida et al., 2022), and neurophysiology training guidelines (Björn et al., 2020). Samples of EEG signals were chosen either to be representative of a certain context (Du Pont-Thibodeau et al., 2017, Lalgudi Ganesan et al., 2018) or aimed to provide a broad sampling of common EEG patterns (Ahrens et al., 2021, Amorim et al., 2017, Asukile et al., 2022, Leira et al., 2004). Three of the studies clearly stated that the test was not validated (Gilbert et al., 2000, Swarnalingam et al., 2022, Yuan et al., 2022). Two of the studies reported validity testing using experts (Björn et al., 2020, Linnavuori et al., 2022 Linnnavuori et al., 2022). One study ensured validity by using an expert to evaluate the questions with a suitable difficulty level beforehand (Björn et al., 2020). Another study used an expert panel to assess the content validity of the instrument by using 6 experts to assess each item's relevance, importance, and clarity. The instrument was also pre-tested among the target population (n = 3) (Linnavuori et al., 2022). Internal consistency was assessed in two studies by calculating the Cronbach α coefficient that varied between 0.829 and 0.956 (Linnavuori et al., 2022) or by repeating two questions in their exact form confirming internal consistency (Asukile et al., 2022).

## 5 Discussion

The purpose of this review was to describe the EEG competence of health professionals and how it has been measured in previous literature. The results provide information on EEG competence and future assessment needs. Based on the results EEG competence was found to consist of knowledge, skills, attitude, and values which can then be divided into different EEG components that healthcare professionals are expected to have. In addition, various individual and work-related factors were identified that influence the competence levels of these EEG components. However, EEG competence has been measured in different ways, creating the need to develop a validated instrument to assess competence comprehensively in the future.

The review revealed that the concept of EEG competence has not yet been fully developed. Based on the results, EEG competence consists of different components and these components correspond to earlier studies (Linnavuori et al., 2022). Although the aim was to find as many studies on EEG competence as possible, the search terms with different competence attributes may have limited the search results and therefore perhaps not all components of EEG competence were identified. Furthermore, most of the studies only focused on EEG interpretation in ICU settings highlighting the specificities of intensive care and neglecting other important areas of EEG competence. In the future, clearly defining EEG competence will be important so as to establish a basis for an instrument to measure EEG competence. Currently, tools to assess EEG competence of healthcare professionals were developed for each single study and there was a lack of validated instruments to measure EEG competence comprehensively; this makes it difficult to compare competence results. To measure the competence of health professionals, it would be useful to use both an analytical and a holistic approach to obtain an overall view of competence (Rotthoff et al., 2021). The results of this review revealed different methods for both approaches, which can be used in the future.

The result of this review highlights that nurses are also expected to have EEG competence even though it is not part of their curricula. (Cole et al., 2022). Nurses are already monitoring and interpreting patients' vital signs and EEG competence may become one of these skills in the future (Legriel et al., 2021). For EEG professionals such as EEG technicians and biomedical laboratory scientists, this may mean taking greater responsibility for teaching and monitoring the quality of diagnostic tests in their specialty. More attention should be paid to this collaboration and the clarification of the roles of these healthcare professionals in the future. In addition, future research should focus on identifying the factors that affect EEG competence because education in this field seems to vary. This review identified various factors, but more research is needed. Individual-related factors such as work experience were associated with self-assessed EEG competence (Linnavuori et al., 2022) but these factors were not associated with objectively assessed competence e.g., the interpretation of the EEG (Ahrens et al., 2021).

Most of the studies included focused on healthcare professionals’ competence in interpreting EEG. The ability to interpret results is also emphasized in other diagnostic tests such as electrocardiograms (ECG) (Chen et al., 2022) and x-rays (Kearns et al., 2024). However, the requirements for EEG technicians to interpret the EEG signal vary (Cole et al., 2022). It is also important to consider how these interpretations are used in practice. Usually, interpretations of test results by nurses and technicians are used to alert EEG professional physicians (e.g., Bourgoin et al., 2020). However, if the physician is not available and the interpretation is used as a basis for seizure treatment and administering medicines, this may also raise legal issues. Therefore, the optimal role of healthcare professionals in EEG interpretation requires further research to inform future policy discussions.

However, it is important to note that competence in interpreting an EEG is not enough to make a reliable diagnosis. In the total testing process, pre-analytical errors are the most common (Hawkins 2012) which should also be taken into account in the EEG because improperly conducted EEG recording can lead to diagnostic errors. In the future, it will be important to understand the total EEG testing process to ensure the quality of the test results. When interpreting an EEG, the importance of understanding the basic biophysical aspects of signal generation and recording technology is highlighted (Beniczky & Schomer 2020). This is also important for healthcare professionals who are expected to undertake an EEG. This is also underlined by the fact that in the future EEG technology is developing (Herman et al., 2015), and various automated EEG analysis tools are being developed (Wong et al., 2023). Therefore, due to advancing technology, knowledge and skills are important to identify potential issues during the testing process, such as poor-quality data, artifacts, or technical failures, and take corrective actions. In this study, collaboration with the patient and other healthcare professionals was also identified as an area of EEG competence, which has been found in previous studies to be an important part of preventing diagnostic errors (Olson et al 2019).

### 5.1 Limitations

The limitations of this review are related to the search strategy and analysis of the studies. Although the search strategy was developed with the assistance of an information specialist, and the search strategy produced a large number of search results, many of these were duplicates between databases or were excluded in the screening phase. Any selection bias was reduced by two researchers screening the studies and then discussing disagreements between the reviewers. In defining the keywords, the aim was to use as wide a range of keywords as possible in relation to the different attributes of competence. In addition to these, common terms in use for EEG interpretation were used. However, not all the studies used these attributes of competence. The aim of this review was to search for articles from four databases that had empirically examined healthcare professionals’ EEG competence. Only studies that measured EEG competence were selected for inclusion. This may have biased the analysis, as most of the studies focused on EEG interpretation competence. In addition, no grey literature or unpublished studies were included. This may lead to reporting bias due to missing results. The results of this review are also limited by the fact that some studies did not use validated instruments to measure EEG competence or did not always adequately describe the measurement properties of these instruments. None of the included studies used a reporting checklist (e.g. Consensus-based Standards for the Selection of Health Measurement Instruments, COSMIN). Therefore, not all parts of the measurement tools were necessarily identified, or how the tool was developed.

## 6 Conclusions

Although increasing attention is being paid to the EEG competence of healthcare professionals, it has rarely been measured comprehensively and there is no validated instrument that has been tested for psychometric properties. Therefore, a valid assessment instrument to comprehensively measure EEG competence will be needed. The EEG competence expected of healthcare professionals includes knowledge and skills as well as attitudes and values, which consist of several different components including: interpreting EEG, having a theoretical knowledge base in the field, managing, and using medical devices, collaborating among patients and other professionals, patient care, confidence to act and comfort with own EEG competence. In the future, it will be important to take these into account when defining and assessing EEG competence. Some individual and work-related factors were identified that influence EEG competence, but further research is needed to confirm these findings. Addressing these gaps is crucial for enhancing the proficiency in EEG of healthcare professionals and, consequently, improving patient care.

## CRediT authorship contribution statement

**Elina Linnavuori:** Conceptualization, Data curation, Methodology, Investigation, Writing – original draft, Writing – review & editing. **Irina Virtanen:** Conceptualization, Investigation, Project administration, Supervision, Writing – review & editing. **Minna Stolt:** Conceptualization, Project administration, Supervision, Writing – review & editing.

## Declaration of Competing Interest

The authors declare that they have no known competing financial interests or personal relationships that could have appeared to influence the work reported in this paper.

## Data Availability

Data will be made available on request.
