# Supplementary material for: Competence of healthcare professionals performing electroencephalography test: A systematic review
Source: Clin Neurophysiol Pract. 2025 Mar 8;10:104–15. doi: 10.1016/j.cnp.2025.03.001 (PMC11951942; doi:10.1016/j.cnp.2025.03.001)
Supplement: Supplementary Data 1 [file mmc1.docx]

Suplementary file 1. Mixed methods appraisal tool (MMAT) quality evaluation (Hong et al. 2018)

| Studies (n=28) | Methodological quality criteria | | | | |
| --- | --- | --- | --- | --- | --- |
| **Qualitative studies** | **1.1** | **1.2** | **1.3** | **1.4** | **1.5** |
| Beirne et al. 1996 | yes | yes | no | yes | yes |
| **Quantitative non-randomized studies** | **3.1** | **3.2** | **3.3** | **3.4** | **3.5** |
| Amorim et al. 2017 | no | yes | no | yes | yes |
| Asukile et al. 2022 | yes | yes | yes | yes | yes |
| Beuchat et al. 2021 | no | yes | yes | yes | yes |
| Björn et al. 2020 | no | no | yes | no | yes |
| Bourgoin et al. 2020 | no | yes | yes | no | yes |
| Dericioglu et al. 2015 | no | yes | yes | yes | yes |
| Du Pont-Thibodeau et al. 2017 | no | yes | yes | no | yes |
| Ganesan et al. 2018 | no | yes | yes | yes | yes |
| Gilbert et al. 2000 | yes | no | yes | yes | yes |
| Goswami et al. 2018 | yes | yes | yes | no | yes |
| Kaleem et al. 2021 | yes | yes | yes | no | yes |
| Kang et al. 2019 | no | yes | no | no | yes |
| Kolls et al. 2012 | no | yes | no | no | yes |
| Legriel et al. 2021 | yes | no | yes | no | yes |
| Leira et al. 2004 | yes | yes | yes | no | yes |
| Mehta et al. 2017 | no | yes | yes | yes | yes |
| Ouchida et al. 2022 | no | no | yes | yes | yes |
| Picinich et al. 2020 | yes | no | yes | no | yes |
| Poon et al. 2015 | no | yes | yes | yes | yes |
| Prendergast et al. 2022 | yes | no | yes | no | yes |
| Seiler et al. 2012 | yes | no | no | yes | yes |
| Swarnalingam et al. 2022 | yes | yes | yes | no | yes |
| Swisher et al. 2015 | no | yes | yes | no | yes |
| Topjian et al. 2015 | no | yes | no | yes | yes |
| Yuan et al. 2022 | yes | no | no | yes | yes |
| **Quantitative descriptive studies** | **4.1** | **4.2** | **4.3** | **4.4** | **4.5** |
| Ahrens et al. 2021 | no | no | yes | yes | yes |
| Linnavuori et al. 2022 | yes | yes | no | yes | yes |
